# The New One Board Bill Medical Examiners for Texas

**Published:** 1907-03

**Authors:** 


### Abstracts and Selections

### The New One Board Bill Medical Examiners for Texas.

A BILL

TO BE ENTITLED

*An Act to repeal Chapter 12 of the General Laws of Texas, passed by the Twenty-seventh Legislature, page 12, Laws of 1901, and to pass in lieu thereof this act; to create a Board of Medical Examiners for the examination and licensing of all physicians, surgeons, and obstetricians; to prescribe their qualifications; to provide for their proper registration, the revocation of their licenses for flagrant offenses; and to fix suitable penalties for illegal practice.*

Be it enacted by the Legislature of the State of Texas:

Section 1. That a board, to be known as the Board of Medical Examiners for the State of Texas, is hereby established. Said board shall consist of eleven men learned in medicine, legal and active practitioners in the State of Texas, who shall have resided and practiced medicine in this State under a diploma from a legal and reputable college of medicine of the school to which said practitioner shall belong for more than three years prior to their appointment, and no one school shall have a majority representation on said board. Said board shall be appointed by the Governor of this State within ninety days after his inauguration, and the term of office of its members shall be two years, or until their successors shall be appointed and qualified. Each regular organized State association of practicing physicans that comes under the provisions of this act shall furnish the Governor ten names from each such State association of practicing physicians, from whom the appointments of this board shall be selected. No member of said board shall be a stockholder or a member of the faculty or a board of trustees of any medical school. Vacancies occurring in the board shall be filled by the Governor. The word "medicine" as used in this section shall have the same meaning and scope as given to it in Section 13 of this act.

Sec. 2. The members of said board shall qualify by taking the oath of office before a notary public or other officer empowered to administer oaths in the county in which each shall respectively reside. At the first meeting of said board after each biennial appointment the board shall elect a president, vice-president and secretary-treasurer. Six members shall constitute a quorum. Regular meetings shall be held at least twice a year, at such times and places as shall be deemed most convenient for applicants. Due notice of such meetings shall be given by publication in such papers as may be selected by the board. Special meetings may be held upon a call of three members of the board. The board may prescribe rules, regulations and by-laws, in harmony with the provisions of this act, for its own proceedings and government for the examination of applicants for the practice of medicine and obstetrics. Said board, or any member, shall have power to administer oaths for all purposes required in the discharge of its duties, and to adopt a seal to be affixed to all of its official documents.

Sec. 3. The Board of Examiners shall preserve a record of its proceedings in a book kept for that purpose, showing name, age, place and duration of residence of each applicant, the time spent in medical study in respective medical schools, and the year and school from which degrees were granted; said register shall also show whether applicants were rejected or licensed, and shall be prima facie evidence of all matters contained therein. The secretary of the board shall, on March 1 of each year, transmit an official copy of said register to the Secretary of State for permanent record, certified copy of which, with hand and seal of the secretary of said board, or Secretary of State, shall be admitted in evidence in all courts.

Sec. 4. From and after the passage of this act it shall be unlawful for any one to practice medicine in any of its branches upon human beings within the limits of this State who has not registered in the district clerk's office of the county in which he resides, his authority for so practicing, as herein prescribed, together with his age, postoffice address, place of birth, school of practice to which he professes to belong, subscribed and verified by oath, which, if wilfully false, shall subject the applicant to conviction and punishment for false swearing as provided by law. The fact of such oath and record shall be indorsed by the district clerk upon the certificate. The holder of the certificate must have the same recorded upon each change of residence to another county, and the absence of such record shall be prima facie evidence of the want of possession of such certificate.

Sec. 5. It is hereby made the duty of the district clerk of each county in this State to purchase a book of suitable size, to be known as the "Medical Register" of such county, and set apart one full page for the registration of each physician, and to record in the same the name and record of each practitioner who presents a certificate from the State Board of Examiners, issued under this act. The clerk shall receive the sum of one dollar from each physician so registered, which shall be his full compensation for all duties required under this act. When any physician shall die or remove from the county, or have his license revoked, it shall be the duty of said clerk to make a note of facts at the bottom of the page as closing the record. On the 1st day of January in each year said clerk shall, on request of the board, certify to the office of the State Board of Medical Examiners a correct list of the physicians then registered in the county, together with such other information as said board may require. Any district clerk, upon conviction of knowingly violating any of the provisions of this act, shall be fined not more than $50. A copy from the medical register pertaining to any person certified to by said clerk under the seal of said court; also a certificate issued by said officer certifying that any person named has or has not registered in said office as required by this act, shall be admitted in evidence in all trial courts.

Sec. 6. Within one year after the passage of this act all legalized practitioners of medicine in this State, who, practicing under the provisions of previous laws, or under diplomas of a reputable and legal college of medicine, have not already received license from a State Medical Examining Board of this State, shall present to the Board of Medical Examiners for the State of Texas documents, or legally certified transcripts of documents, sufficient to establish the existence and validity of such diplomas or of the valid and existing license heretofore issued by previous examining boards of this State, or exemption existing under any law, and shall receive from said board verification license, which shall be recorded in the district clerk's office in the county in which the licentiates may reside. Such verification license shall be issued for a fee of 50 cents to all practitioners who have not already received a license from a State Board of Medical Examiners of this State. It is especially provided that those whose claims to State licenses rest upon diplomas from medical colleges recorded from January 1, 1891, to July 9, 1901, shall present to the State Board of Medical Examiners satisfactory evidence that their diplomas were issued from bona fide medical colleges of reputable standing, which shall be decided by the Board of Medical Examiners before they are entitled to a certificate from said board. This board may in its discretion, arrange for reciprocity in license with the authorities of other States and Territories having requirements equal to those established by this act. License may be granted applicants for license under such reciprocity on payment of $20.

Sec. 7. All applicants for license to practice medicine in this State who are not licensed under the provisions of the previous section must successfully pass an examination before the Board of Medical Examiners established bv this act. Applicants to be eligible for examination must present satisfactory evidence to the board that they are more than 21 years of age, of good moral character, and graduates of bona fide, reputable medical schools. Such schools shall be considered reputable within the meaning of this act whose entrance requirements and courses of instruction are as high as those adopted by the better class of medical schools of the United States, to be determined and decided by the Board of Medical Examiners. Application for examination must be made in writing under affidavit to the secretary of the board, on forms prepared by the board, accompanied by a fee of $15; except when an applicant desires to practice obstetrics alone the fee shall be $5. Such applicants shall be given due notice of the date and place of examination. Applicants to practice obstetrics in the State of Texas, upon proper application, shall be examined by the board in obstetrics only, and upon satisfactory examination shall be licensed to practice that branch only; provided, this shall not apply to those who do not follow obstetrics as a profession, and who do not advertise themselves as obstetricians or midwives, or hold themselves out to the public as so practicing. In case any applicant, because of failure to pass examination, be refused a license, he or she shall, after one year, be permitted to take a second examination without an additional fee.

Sec. 8. The fund realized from the aforesaid fees shall be applied first to the payment of necessary expenses of the Board of Examiners; any remaining funds shall be applied by the order of the board to compensating members of board in proportion to their labors.

Sec. 9. All examinations shall be conducted in writing and in such manner as shall be entirely fair and impartial to all individuals and every school of medicine, the applicants being known by numbers, without names or other method of identification on examination papers by which members of the board may be able to identify such papers, until after the applicants have been granted licenses or rejected. Examinations shall be conducted on the scientific branches of medicine only, and shall include anatomy, physiology, chemistry, histology, pathology, hygiene, and medical jurisprudence. Upon satisfactory examination under the rules of the board, applicants shall be granted licenses to practice medicine. All questions and answers, with grades attached, shall be preserved for one year. All applicants examined at the same time shall be given identical questions in each of the above branches. All certificates shall be attested by the seal and signed by all members of the board.

Sec. 10. Nothing in this act shall be construed as to discriminate against any particular school or system of medical practice. This act shall not apply to dentists legally qualified and registered under the laws of this State who confine their practice strictly to dentistry; nor to nurses who practice only nursing; nor to masseurs, in their particular sphere of labor, who publicly represent themselves as such; nor to commissioned or contract surgeons of the United States Army, Navy or Public Health and Marine Hospital Service, in the performance of their duties, but such shall not engage in private practice without license from the Board of Medical Examiners; nor to legally qualified physicians of other States called in consultation, but who do not open offices or appoint places in this State where patients may be met or called to see. This act shall be so construed as to apply to persons other than licensed druggists of this State not pretending to be physicians, who offer for sale on the streets or other public places remedies which they recommend for the cure of disease.

Sec. 11. The State Board of Medical Examiners may refuse to admit persons to its examinations or to issue the certificate provided for in this act for any of the following causes:

First. The presentation to the board of any license, certificate or diploma which was illegally or fraudulently obtained, or when fraud or deception has been practiced in passing the examination.

Second. Conviction of a crime of the grade of a felony, or one which involves moral turpitude, or procuring, or aiding or abetting the procuring of a criminal abortion.

Third. Other grossly unprofessional or dishonorable conduct of a character likely to deceive or defraud the public; or for habits of intemperance or drug addiction calculated to endanger the lives of patients; provided, that any applicant who may be refused admittance to examination before said board shall have the right of action to have such issue tried in the district court of the county in which some member of the board shall reside.

Sec. 12. The right herein to practice medicine in this State may be revoked by any court of competent jurisdiction, upon proof of the violation of the law in any respect in regard thereto, or for any cause for which the State Board of Medical Examiners is authorized to refuse to admit persons to its examination as provided in Section 11 of this act; and it shall be the duty of the Board of Medical Examiners and of any member thereof to institute such suit, in the name of the State, upon the relation of such board or any such member. Such action shall be in the nature of a quo warranto, and shall be governed, as near as practicable, by the law and rules relative thereto.

Sec. 13. Any person shall be regarded as practicing medicine within the meaning of this act who shall publicly profess to be a physician or surgeon or shall treat or offer to treat any disease or disorder, mental or physical deformity or injury, by any system or method, or to effect cures thereof and charge therefor, directly or indirectly, money or other compensation.

Sec. 14. Any person practicing medicine in this State, as defined in this act, in violation of the provisions of this act, shall, upon conviction, be fined not less than $50 nor more than $500, and by imprisonment not exceeding six months in the county jail, and in no case where any provision of this act is violated shall the violator be entitled to recover by action, suit or warrant, any compensation for the service rendered, and each day shall constitute a separate offense.

Sec. 15. All certificates heretofore issued by medical examining boards under former laws are hereby recognized as in full force, and are hereby confirmed, and are subject to the provisions of this act as though issued under it.

Sec. 16. Any person practicing medicine in this State, who shall procure from another any money, property, obligation, contract or other thing of value by making any false or fraudulent representation, statement or promise, and fails or refuses to return such money, porperty, obligation, contract or other thing of value to the person from whom it was so procured, shall be guilty of a misdemeanor, and, upon conviction, shall be fined in any sum not less than $100 nor more than $500, and such person may be tried or prosecuted in the county where the person so defrauded resides, and in addition thereto a judgment may be rendered in said case forfeiting the license of such physician to practice medicine.

Sec. 17. All laws and parts of laws in conflict with the provisions of this act be and the same are hereby repealed.

Sec. 18. The fact that there is now no law properly regulating the practice of medicine in this State creates an emergency and an imperative necessity that the constitutional rule requiring bills to be read on three several days be suspended, and the same is hereby suspended, and that this act take effect and be in force from and after its passage, and it is so enacted.

